# *Mycobacterium tuberculosis* Rv3402c Enhances Mycobacterial Survival within Macrophages and Modulates the Host Pro-Inflammatory Cytokines Production via NF-Kappa B/ERK/p38 Signaling

**DOI:** 10.1371/journal.pone.0094418

**Published:** 2014-04-10

**Authors:** Wu Li, Quanju Zhao, Wanyan Deng, Tian Chen, Minqiang Liu, Jianping Xie

**Affiliations:** Institute of Modern Biopharmaceuticals, State Key Laboratory Breeding Base of Eco-Environment and Bio-Resource of the Three Gorges Area, Key Laboratory of Ministry of Education Eco-Environment of the Three Gorges Reservoir Region, School of Life Sciences, Southwest University, Chongqing, China; University of Malaya, Malaysia

## Abstract

Intracellular survival plays a central role in the pathogenesis of *Mycobacterium tuberculosis*, a process which depends on an array of virulence factors to colonize and replicate within the host. The *M. tuberculosis* iron regulated open reading frame (ORF) *rv3402c*, encoding a conserved hypothetical protein, was shown to be up-regulated upon infection in both human and mice macrophages. To explore the function of this ORF, we heterologously expressed the *rv3402c* gene in the non-pathogenic fast-growing *Mycobacterium smegmatis* strain, and demonstrated that Rv3402c, a cell envelope-associated protein, was able to enhance the intracellular survival of recombinant *M. smegmatis*. Enhanced growth was not found to be the result of an increased resistance to intracellular stresses, as growth of the Rv3402c expressing strain was unaffected by iron depletion, H_2_O_2_ exposure, or acidic conditions. Colonization of macrophages by *M. smegmatis* expressing Rv3402c was associated with substantial cell death and significantly greater amount of TNF-α and IL-1β compared with controls. Rv3402c-induced TNF-α and IL-1β production was found to be mediated by NF-κB, ERK and p38 pathway in macrophages. In summary, our study suggests that Rv3402c delivered in a live *M. smegmatis* vehicle can modify the cytokines profile of macrophage, promote host cell death and enhance the persistence of mycobacterium within host cells.

## Introduction

Tuberculosis (TB), caused by the facultative intracellular pathogen *Mycobacterium tuberculosis* (Mtb), remains a major cause of morbidity and mortality around the world. Despite intense efforts to mitigate its toll on humanity, TB was still responsible for 1.3 million deaths, and caused 8.6 million new and relapse infections in 2012 (World Health Organization Tuberculosis Data and Statistics, 2013). In addition, the emergence of drug resistant Mtb strains and co-infection with HIV further highlighted the necessity of novel TB medications informed by in-depth understanding of Mtb biology.

Upon internalization by macrophages, Mtb is widely assumed to be confined within the phagosome, a specialized vacuole derived from the plasma membrane. Subsequently, the phagosome matures through fusion with late endosomes and lysosomes, creating an inhospitable environment for invading microorganisms, that includes phagosomal acidification, elevated levels of reactive oxygen intermediates (ROI) and reactive nitrogen intermediates (RNI), charged antimicrobial peptides [1] and iron deprivation [2]. However, Mtb has evolved a spectrum of subversive strategies to survive and thrive inside the macrophages [3].

The success of Mtb can be attributed to an array of virulence factors. These effectors can be classified as (a) enhancing the resistance to host toxic compounds, such as KatG [4], SodC [5], [6], AhpC [7], [8], (b) blocking phagosomes maturation, such as PtpA [9], [10], PknG [11], [12], SapM [13], [14], and (c) evading apoptosis through NuoG [15], [16], [17] and SodA [18], [19]. The identification and characterization of novel virulence factors can enrich our understanding of Mtb biology and facilitate better control measures.

Iron scavenging capacity is critical for pathogen proliferation and pathogenesis within host [20]. The Mtb ORF *rv3402c*, encoding an IdeR- and iron-repressed gene, was found to be significantly upregulated during Mtb growth under iron-limiting conditions [21]. Interestingly, *rv3402c* was further shown to be induced in human THP-1 [21] and primary bone marrow-derived murine macrophages [22], suggesting a role in the adaptation to intracellular niches.

In the following study, we investigated the role of Rv3402c through the construction of recombinant *M. smegmatis* expressing strains. We discovered that Rv3402c was able to enhance *M. smegmatis*' intracellular survival as well as alter the cytokine profile of infected macrophages.

## Materials and Methods

### Bacterial strains and growth conditions

*Escherichia coli* and *M. smegmatis* mc^2^ 155 strains were provided by the Institute of Modern Biopharmaceuticals. Mtb H37Rv genomic DNA was provided by Chongqing Pulmonary Hospital. The human leukemic monocyte lymphoma cell line (U-937) was purchased from the Conservation Center in Wuhan University (China), and the murine macrophage cell line (RAW264.7) was a kind gift from Zhiren Zhang [23] (Third Military Medical University, China). *Escherichia coli* DH5α was routinely grown in LB medium for its use in DNA cloning procedures. *M. smegmatis* mc^2^ 155 was grown at 37°C in Middlebrook (MB) 7H9 liquid medium or on MB 7H10 agar supplemented with 0.2% (w/v) glucose, 0.5% (v/v) glycerol and 0.05% (v/v) Tween 80. When required, kanamycin (20 μg/ml) or hygromycin (50 μg/ml) was added.

### Gene amplification, plasmids construction, and recombinant *M. smegmatis*

The primers used in this study are listed in Table 1. The pNIT-1 plasmid used in this study has been previously described [24]. Briefly, pNIT-Myc was constructed from the pNIT-1 mycobacterial shuttle vector harboring a Myc-tag to the C-terminal of Rv3402c in order to perform Western blot. The full length *rv3402c* gene was amplified from Mtb H37Rv genomic DNA. Amplified *rv3402c* gene was then cloned into the pNIT-Myc vector. Constructs for the plasmid pALACE-Rv3402c (at the N terminus) were made by using the similar method as pNIT-Myc-Rv3402c. The *gfp* gene was excised from pSC301 [25] by digesting with *BamHI* and *ClaI*, then cloned into pALACE digested with the same restriction enzymes. The plasmids were electroporated into *M. smegmatis* mc^2^ 155 according to standard procedures [26]. The recombinant *M. smegmatis* strains were selected on MB 7H10 agar containing kanamycin (for pNIT-Myc) or hygromycin (for pALACE). Gene expression was confirmed by PCR, and the recombinant strains were stored at −70°C until further use.

**Table 1:** Primers used in this study.

	Sequence
*Cloning primers*	
pNIT-Rv3402c-For	CGCGGAATTCATGAAGATCCGAAC
pNIT-Rv3402c-Rev	AATGGATCCTTCACCGCGCACCT-3
pALACE-Rv3402c-For	GAGGGATCCATGAAGATCCGAACGTT
pALACE-Rv3402c-Rev	ACACATATGTCATTCACCGCGCACC
pET-28-Rv3402c-For	CGCTGGATCCATGAAGATCCGAAC
pET-28-Rv3402c-Rev	GAGAAGCTTTCATTCACCGCGCAC
*RT-PCR primers*	
pNIT-Rv3402c-For	ACCGCTACCTGCTGATG
pNIT-Rv3402c-Rev	GGATGGACTCGCGTGTTTG
16 S rRNA-For	GTAGGGGAAAGCTTTTGCGGTGTGG
16 S rRNA-Rev	TCGTCTGTGCTGAAAGAGGTTTACA
TNF-α-For	CGCTCCCCAAGAAGACAG
TNF-α-Rev	TGAAGAGGACCTGGGAGT
IL-1 β-For	GATGGCTTATTACAGTGGC
IL-1 β-Rev	GACCAGACATCACCAAGC
β-actin-For	CGGCTCCGGCATGTGCAA
β-actin-Rev	ATGTCACGCACGATTTCC

### Expression of Rv3402c in *M. smegmatis*

The recombinant *M. smegmatis* strains expressing Myc-tagged Rv3402c (MS_Rv3402c) and the empty pNIT-Myc vector (MS_Vec) were cultured until an OD_600_ 0.6–0.8 was reached. Protein expression was induced with 28 mM ε-caprolactam (Aladdin, China). Total bacterial RNA was isolated using RNAprep Pure Cell/Bacteria Kit (TIANGEN, China) after 16 h induction. An equal amount of total RNA was used as a template for reverse transcriptase (RT)-PCR with *rv3402c* specific primes and mycobacterium 16 S rRNA (*rrsB* gene) specific primers. The RT-PCR products were visualized using a 1% agarose gel.

For the detection of Myc-tagged Rv3402c bacterial pellets were harvested and disrupted by ultrasonication. Samples were subjected to SDS-PAGE and the Myc-tagged Rv3402c protein was detected by the mouse anti-Myc antibody (TIANGEN, China).

### Subcellular fractionation of *M. smegmatis*

Subcellular fractionation was performed as described by Bashiri *et al*. [27], with minor modifications. In short, 200 ml of *M. smegmatis* was grown to an OD_600_ ∼0.8 and was induced with 28 mM ε-caprolactam. Next, the cells were harvested, resuspended in 9 ml lysis buffer (PBS, 1 mM PMSF, 0.6 mg/ml of both DNase and RNase) and then lysed by ultrasonication. Lysates were centrifuged at 3,000 g for 30 min to obtain the whole cell lysates (WCL) from the supernatant. WCL was centrifuged at 27,000 g for 30 min to obtain the cell wall pellet (CW). The supernatant from the WCL fraction was further centrifuged at 100,000 g for two hours to separate the cell membrane fraction (pellet, CM) from the soluble fraction (supernatant, SOL). CW and CM fractions were washed once with lysis buffer, re-centrifuged and subsequently re-suspended in 0.5 mL of lysis buffer. All centrifugation steps were performed at 4°C.

The above fractionated samples were subjected to SDS-PAGE, with the ratio of protein concentrations at 1∶0.36∶0.71∶0.91 (WCL∶CW∶CM∶SOL). After separation, proteins were transferred to a nitrocellulose membrane. The Myc-tagged Rv3402c protein was detected by the mouse anti-Myc antibody. Native *M. smegmatis* GroEL2, which contains a string of endogenous histidines [28], was detected by an anti-His mouse primary antibody (TIANGEN). The secondary antibody used was goat anti-mouse conjugated to HRP (TIANGEN). The signal was detected using Western Lighting ECL.

### Proteinase K and trypsin sensitivity assays

Proteinase K and trypsin sensitivity assays were performed as previously described [29]. Briefly, *M. smegmatis* harboring pALACE, pALACE-Rv3402c or pALACE-GFP were induced with 0.2% acetamide for 10 h and harvested. Cells were washed and mixed with proteinase K or trypsin at a concentration of 100 μg/ml and incubated at 37°C for the indicated times. The reaction was stopped by adding 100 nM PMSF. The samples were dissolved in SDS loading buffer and analyzed by Western blotting using anti-His mouse primary antibody (Abm Inc.) or anti-GFP mouse primary antibody (Boehringer Mannheim Corp.). The secondary antibody used was Alexa Fluor 660 Goat Anti-Mouse IgG (Life technologies Corp.). The signal was detected using Odyssey CLx Infrared Imaging System.

### Purification of recombinant Rv3402c protein

Recombinant Rv3402c (rRv3402c) was expressed in *E. coli* according to a published protocol [30]. Briefly, the full length *rv3402c* gene was cloned into the pET-28a(+) vector and the protein was purified by His-tag affinity chromatography on a Ni^2+^ - nitrilotriacetate column. Purified rRv3402c was dialyzed against PBS (pH 7.2), and then applied to an E-TOXATE Kit (Sigma, USA) to remove any endotoxin contamination prior to filter-sterilization, and then stored at −70°C until further use.

### Intracellular survival assay

Suspension cultures of U-937 cells were seeded at 1×10^6^ cells per well in 12-well tissue culture plates. Following 48 hours of treatment with 0.1 mg/ml of phorbol 12-myristate 13-acetate (PMA) (Sigma), U-937 cells were transformed into an adherent state. Cells were infected with *M. smegmatis* transformants at a MOI of 10∶1 (bacteria-to-U-937 ratio) at 37°C in 5% CO_2_. Four hours after infection, the remaining bacteria in the culture were removed by washing three times with warm RPMI-1640. RPMI 1640–10% FCS containing 250 nM IVN (Sigma) in DMSO and hygromycin B (Roche, USA) (100 μg/ml) were then added. At 6 and 72 h after infection, the culture supernatants were collected, and stored at −70°C. The macrophages were then washed twice and lysed in sterile water containing 0.025% (w/v) SDS. The lysed macrophages were plated on MB 7H10 agar plates containing kanamycin and the colony forming units (CFUs) were determined as a measure of the intracellular survival of recombinant *M. smegmatis*. The intracellular survival assay of *M. smegmatis* in RAW264.7 cells was performed in a similar manner as described for the U-937 cells above. By comparison, RAW264.7 cells were seeded at 5×10^5^ cells per well in 12-well tissue culture plates. Cells were infected with *M. smegmatis* transformants at a MOI of 10∶1 after 24 hours.

### *In vitro* growth and stress assays

For *in vitro* growth curves, cultures were inoculated in triplicates with a starting absorbance (OD_600_) of ∼0.02. Broths were incubated at 37°C with shaking until an OD_600_ of ∼0.8 was reached. Twenty-eight micromolar of ε-caprolactam was added to the cultures, and the OD_600_ was measured at various time points over a 36 h growth period. To assess the growth curve of recombinant *M. smegmatis* in iron-depleted medium, the recombinant strains were grown with 100 μM 2′ 2′ dipyridyl [31]. Growth was monitored by determining the OD_600_ at various time points over 50 h.

To prepare for the pH stress assays [32], cells were harvested, washed with 7H9 (pH 3 or 5) and then resuspended to an OD_600_ of 0.5 in 5 ml 7H9 (pH 3 or 5). Cultures were incubated under stress conditions with agitation and 100 μl samples were removed for viable cell counts after 0, 3 or 6 h. To assess the survival of recombinant *M. smegmatis* after exposure to peroxide stress, the cells were washed and resuspended in 7H9 (pH 5). Five milliliters of each *M. smegmatis* strains, diluted to OD_600_ of 0.5 in 7H9 (pH 5), was exposed to 5 mM H_2_O_2_. At 0 and 6 h, 100 μl samples were removed to determine the viable count. All stress experiments were carried out in triplicates.

### Measurement of LDH release from macrophage

Release of lactate dehydrogenase (LDH) by the U-937 infected with recombinant *M. smegmatis* was measured to evaluate the extent of cytolysis. Culture supernatants were harvested after infection of macrophages with MS_Rv3402c or MS_Vec for 6, 24, 48 or 72 h. LDH activity in culture supernatants was assayed with the CytoTox96 Non-radioactive Cytotoxicity Assay Kit (Promega, USA) as described in the manufacturer's instruction. The percentage of LDH release was calculated according to published methods [33].

### Assay for cytokine production

U-937 cells were infected at 6 and 72 h time points, and processed for analysis by a sandwich ELISA. The concentration of cytokines in the culture supernatant was determined using commercially available ELISA kits for tumor necrosis factor alpha (TNF-α), and interleukin-1 beta (IL-1β) (eBioscience, USA). All assays were performed as recommended by the manufacturer's protocols.

After infection of macrophages with MS_Rv3402c or MS_Vec for 12 h, total RNA was extracted with the RNAprep Pure Cell/Bacteria Kit. The DNAase-treated total RNA (∼1 μg) was transcribed into cDNA with random hexamers using the Transcriptor First Strand cDNA Synthesis Kit (Roche, USA). Semi-quantitative RT-PCR was performed as mentioned above with primers described in Table 1 for the TNF-α, IL-1 β, and β-actin genes.

### Statistical analysis

Data was analyzed using a Student's two-tailed t-test. Statistical significance was defined as a *p*- value of 0.05. Error bars are representative of standard deviation (SD).

## Results

### Expression of *rv3402c* gene in *M. smegmatis*

**Figure 1:**
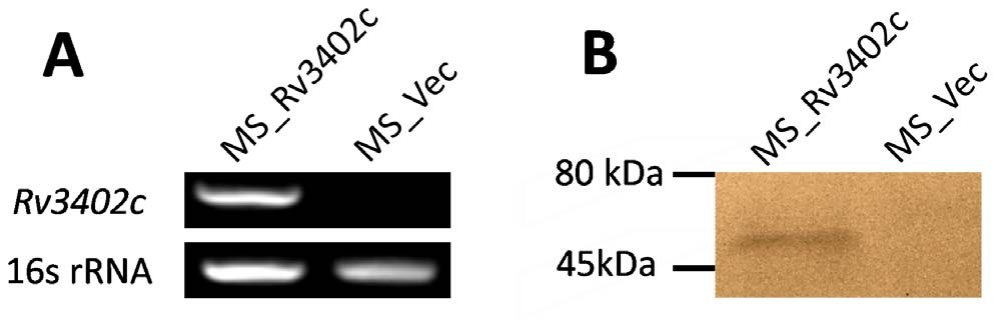
Expression of Rv3402c in recombinant *M. smegmatis*. (A). Ms_Rv3402c and Ms_Vec were grown at 37°C in MB 7H9 liquid medium to an OD_600_ of 0.6–1.0. Total bacterial RNA was isolated after 16 h induction and subjected to RT-PCR to detect expression of the *rv3402c* and *rrsB* genes. (B). Lysates were prepared from bacterial cells that were cultured as in (A) and subjected to Western blot analysis to detect Myc-tagged Rv3402c protein using mouse anti-Myc antibody.

The Mtb ORF *rv3402c* is approximately 1.2 kb in size and encodes for 45 kDa protein. In this study, we generated two recombinant *M. smegmatis* strains to investigate the role of Rv3402c in host interaction. The MS_Rv3402c strain was engineered to express a Myc-tagged Rv3402c protein from a recombinant pNIT-Myc vector, while the MS_Vec strain harbored the vector alone. Both MS_Rv3402c and MS_Vec, which were grown in MB 7H9 medium in the presence of kanamycin, expressed the *aph* gene [34]. The semi-RT-PCR results determined that only MS_Rv3402c was able to express the *rv3402c* gene (Fig. 1A). Western blot analysis using the anti-Myc antibody further confirmed the presence of the expressed ∼50-kDa Rv3402c-Myc fusion protein in the cell lysates of the recombinant strain, and its absence in the parental strain (Fig. 1B). These findings indicate that the Rv3402c protein from *M. tuberculosis* was successfully expressed in *M. smegmatis*.

### Rv3402c is associated with the mycobacterial cell envelope

**Figure 2:**
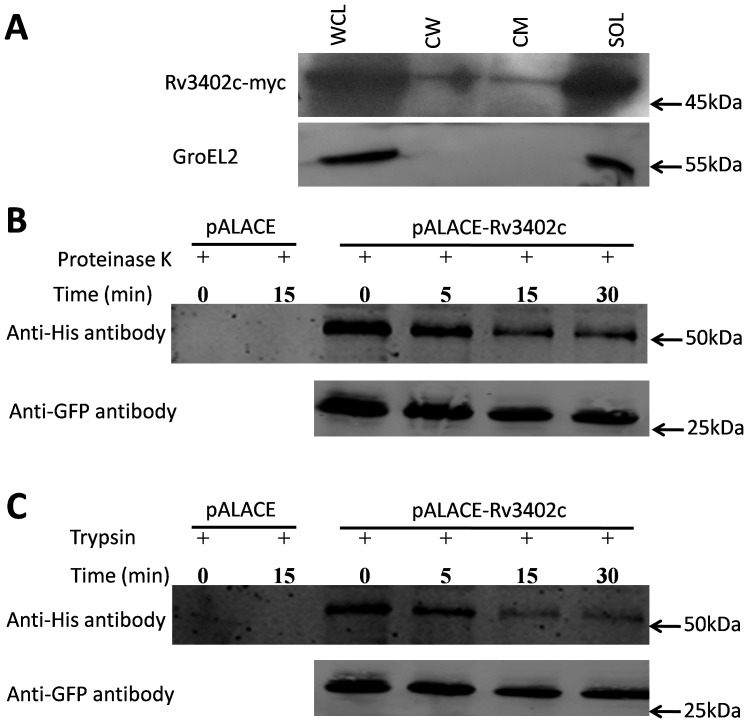
Rv3402c is a cell envelope-associated protein. (A). Subcellular fractionation of *M. smegmatis* induced to express Rv3402c-myc showed localization to the cell wall and membrane fractions. Subcellular fractions were separated by SDS-PAGE and proteins were detected with an anti-Myc antibody. Native GroEL2 was detected as a cytoplasmic control. WCL, whole cell lysates (66 μg total protein); CW, cell wall fraction (24 μg total protein); CM, cell membrane fraction (47 μg total protein); SOL, soluble fraction (60 μg total protein). (B and C). *M. smegmatis* transformed with pALACE, pALACE-GFP or pALACE-Rv3402c were incubated with proteinase K or trypsin at different time points. Whole-cell lysates obtained by the recombinant bacteria were separated on SDS-PAGE. Immunoblots were performed using anti-His antibodies and anti-GFP antibodies. Similar results were obtained in two independent experiments.

Rv3402c is predicted to be engaged in the biosynthesis of lipopolysaccharide (LPS)-like molecules [21]. Moreover, Rv3402c probably involved in cell process, its sequence shows high similarity to the lipopolysaccharide biosynthesis protein from *Bacillus cereus* and a putative PLP-dependent enzyme from *Rheinheimera sp.*, which is predicted to be involved in cell wall biogenesis. We therefore tested whether Rv3402c was present in mycobacterial cell wall and cell membrane fractions. We expressed Myc epitope-tagged Rv3402c in *M. smegmatis*; separated protein extracts into whole cell, cell wall, cytoplasmic, and cell membrane fractions; and analyzed in immunoblot. Although the majority of the Rv3402c proteins were detected in the soluble fraction, small amounts were also seen in the cell wall and cell membrane fractions (Fig. 2A), indicating that Rv3402c is associated with the cell envelope. However, Rv3402c was not detected in culture supernatants and is thus not likely to be secreted extracellularly (data not shown). As controls for fractionation, we show that cytoplasmic heat-shock protein, GroEL2 was detected only in the cytoplasm.

To further confirm the cell surface association of Rv3402c, *M. smegmatis* overexpressing Rv3402c was subjected to proteinase K and trypsin sensitivity assays as previously described [29], [35]. In order to avoid the bias of plasmid pNIT-Myc-Rv3402c, we chose another widely used plasmid pALACE to perform this experiment. As shown in Fig. 2B and C, the His epitope-tagged Rv3402c protein overexpressed by *M. smegmatis* was partly digested by the proteinase K and trypsin treatment, respectively, even the reaction time prolonged to 30 min. Conversely, the recombinant *M. smegmatis* strain expressing only GFP was protected from digestion. Taken together, although the majority of the Rv3402c proteins are localized in the cytoplasm, these results demonstrate that Rv3402c protein is exposed at the mycobacterial cell surface and might gain access to the extracellular environment.

### Rv3402c increases the survival of recombinant *M. smegmatis* in macrophages

**Figure 3:**
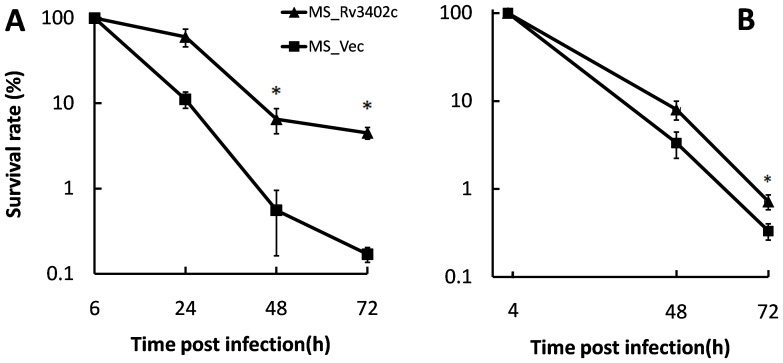
Intracellular survival of recombinant *M. smegmatis* in macrophages. U-937 cells (A) and RAW267.4 cells (B) were infected with MS_Vec and MS_Rv3402c as described in the methods section, respectively. Aliquots of infected cells were lysed with 0.025% SDS at indicated times, and serial dilutions were plated on 7H10 agar plates containing kanamycin. Recovered CFUs were enumerated after the incubation for 3–4 days at 37°C. Numbers of intracellular bacteria are shown as a percentage of the numbers detected at t = 6 h (U-937) or 4 h (RAW267.4) (% survival). Data are shown as means ± SD of triplicate wells. Similar results were obtained in three independent experiments.

Using comparative genomic analysis, no homologus gene could be identified for *rv3402c* in non-pathogenic species such as *M. smegmatis, M. gilvum* and *M. vanbaalenii*. Interestingly, in the attenuated vaccine BCG Pasteur strain, the *rv3402c* ortholog is disrupted into two fragments. One portion of the fragment is annotated as a pseudogene, the other, a 1053 bp gene known as *BCG3472c* (99% identity in DNA sequences). This comparative genomic analysis suggested that *rv3402c* might be a novel virulence factor as this gene is not present in non-pathogenic species. To gain further insight as to whether *rv3402c* is essential for Mtb pathogenesis, we analyzed the intracellular survival of *M. smegamatis* harboring the gene. To achieve this, we infected the mono-layers of U-937 with MS_Vec and MS_Rv3402c as described in the methods section (MOI = 10∶1). The two strains were easily dispersed into single cell suspensions, and the CFU determinations at six hours demonstrated that infection rates were similar in U-937 cells (data not shown). *M. smegmatis* expressing Rv3402c showed significantly higher bacillary counts in U-937 cells at 48 and 72 hours post infection (Fig. 3A), but differences in survival was only observed at 72 h in the RAW264.7 cells (Fig. 3B). Despite these differences, these results suggest that the expression of Rv3402c was able to enhance the intracellular survival of *M. smegmatis* in both human and murine macrophages.

### Enhanced intracellular survival is not correlated with increased resistance to antimicrobial factors

**Figure 4:**
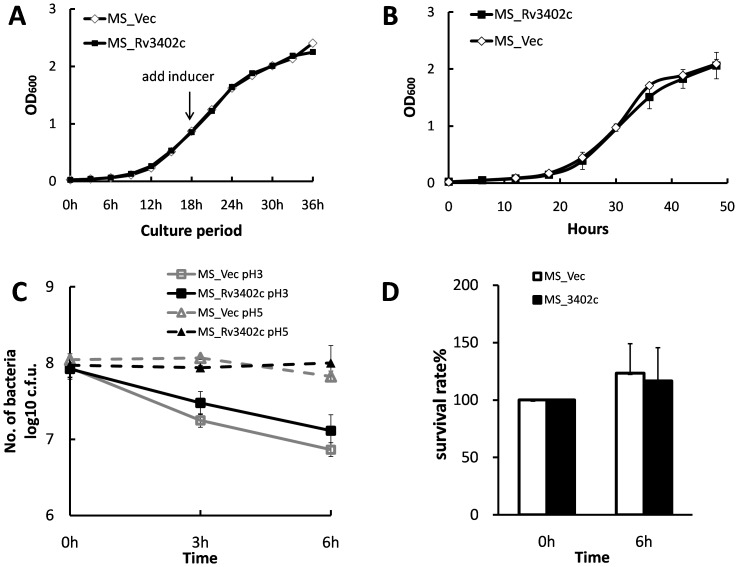
Growth of MS_Vec and MS_Rv3402c under stress conditions. (A). Growth of MS-Vec and MS-Rv3402c at 37°C in MB 7H9 liquid medium was monitored by determining OD_600_ at intervals of 3 h. (B). Growth curve of MS-Vec and MS-Rv3402c in iron-depleted medium. Two recombinant *M. smegmatis* were grown in MB 7H9 medium supplemented with 100 μM 2′ 2′ dipyridyl. The growth of the strains were monitored by measuring OD_600_ at intervals of 6 h. (C). *In vitro* growth of MS-Vec and MS-Rv3402c at different pH. The bacteria were collected by centrifugation and resuspended to an OD_600_ of 0.5 in 5 ml MB 7H9 (pH 5 or 3). All cultures were again incubated at 37°C and 0.1 ml removed for viable count enumeration after 0, 3 and 6 h. (D). Survival of recombinant *M. smegmatis* after exposure to hydrogen peroxide. Aliquots (5 ml) of cultures at OD_600_ of 0.5 were exposed to 5 mM hydrogen peroxide (H_2_O_2_) for 6 h at 37°C. The cultures were serially diluted and plated onto MB 7H10 plates and the colonies counted after 3–4 days of incubation at 37°C.

To gain further insight as to how the *M. smegmatis* strain harbouring *rv3402c* confers greater survival in macrophages, we analyzed growth characteristics between the two strains. As seen in Fig. 4A, the two recombinant strains show the same growth kinetics *in vitro*, suggesting that enhanced survival is likely due to the interaction between mycobacteria and its host. Within the phagosome, the invading microbe is exposed to hostile environment, including iron-deprived conditions, reactive oxygen and reactive nitrogen compounds, and low pH conditions. As a result, we examined whether Rv3402c confers resistance to any of these intracellular stresses.

Given that *rv3402c* is repressed by IdeR and induced by iron limited conditions [21], we explored whether increased survival was due to *M. smegmatis*' resistance to low iron conditions in the macrophage. We compared the ability of the MS_Rv3402c to that of the MS_Vec to grow in MB 7H9 medium in the presence of 2′ 2′ dipyridyl, an iron chelator. No significant differences were observed between the MS_Rv3402c and the empty vector strain in the presence of 100 μM 2′ 2′ dipyridyl (Fig. 4B).

To assess acid sensitivity, growth of the two recombinant *M. smegmatis* strains was monitored in MB 7H9 at pH 3.0 and 5.0, and was compared at three time points during a six hours incubation. No growth differences were observed between these two strains at any time (Fig. 4C). Similarly, susceptibility of the two recombinant strains to ROI was observed following six hours exposure to 5 mM H_2_O_2_. The percentage of survival of MS_Vec and MS_Rv3402c was 123.3% and 116.7%, respectively, with no difference observed between the two recombinants (Fig. 4D). Taken together, these results suggest that the increased survival of MS_Rv3402c in the macrophage cannot be attributed to the increased resistance to any of the tested stresses.

### The effect of the recombinant *M. smegmatis* expressing Rv3402c on the viability of macrophages

**Figure 5:**
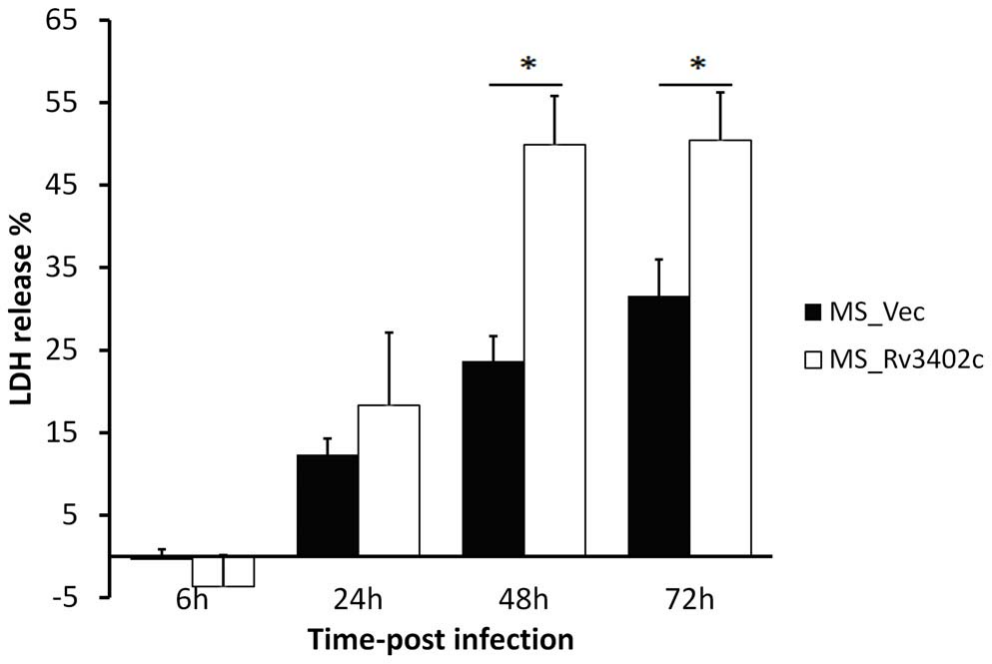
Assay of cell death in macrophages infected with recombinant *M. Smegmatis*. Culture supernatants were collected from mono-layers of U-937 infected at a MOI of 10∶1 with MS_Vec or MS_Rv3402c and the release of LDH as a measure of macrophage cell death was estimated at various time points. Data are shown as means ± SD of triplicate wells. Similar results were obtained in three independent experiments.

One of the destinies of macrophages infected with Mtb is cell death due to the pathogen's ability to alter host cell signaling pathways. To test the effect of Rv3402c on macrophage viability, U-937 cell lines were infected by recombinant *M. smegmatis* containing Rv3402c or the control strain. The amount of LDH was measured in the culture supernatants to quantify cell lysis. Six hours following infection with MS_Rv3402c, there was a significant release of LDH that increased to a range of 49.8% and 50.4% over 72 hours in culture (Fig. 5). In contrast, the percentage of LDH release did not exceed 31% in cultures infected with MS_Vec. These results show that infection with the *M. smegmatis* strain harbouring *rv3402c* was able to induce macrophage lysis.

### Rv3402c upregulates TNF-α and IL-1β production

**Figure 6:**
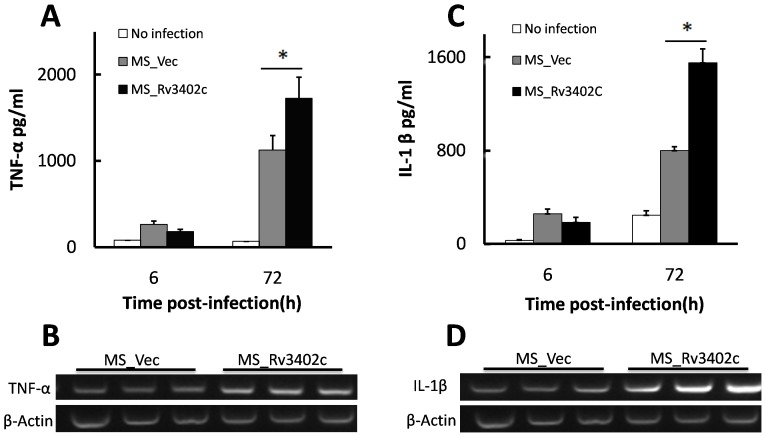
Rv3402c induces the secretion of TNF-α and IL-1β in infected macrophages. Culture supernatants were harvested after 6 or 72-α (A) and IL-1β (C) were determined. Cells were harvested after 12 h of infection and semi-quantitative RT-PCR analysis of TNF-α (B) and IL-1β (D) mRNA level was performed. Each three lanes in the RT-PCR analysis are replicates of a single time point. Similar results were obtained in three independent experiments.

**Figure 7:**
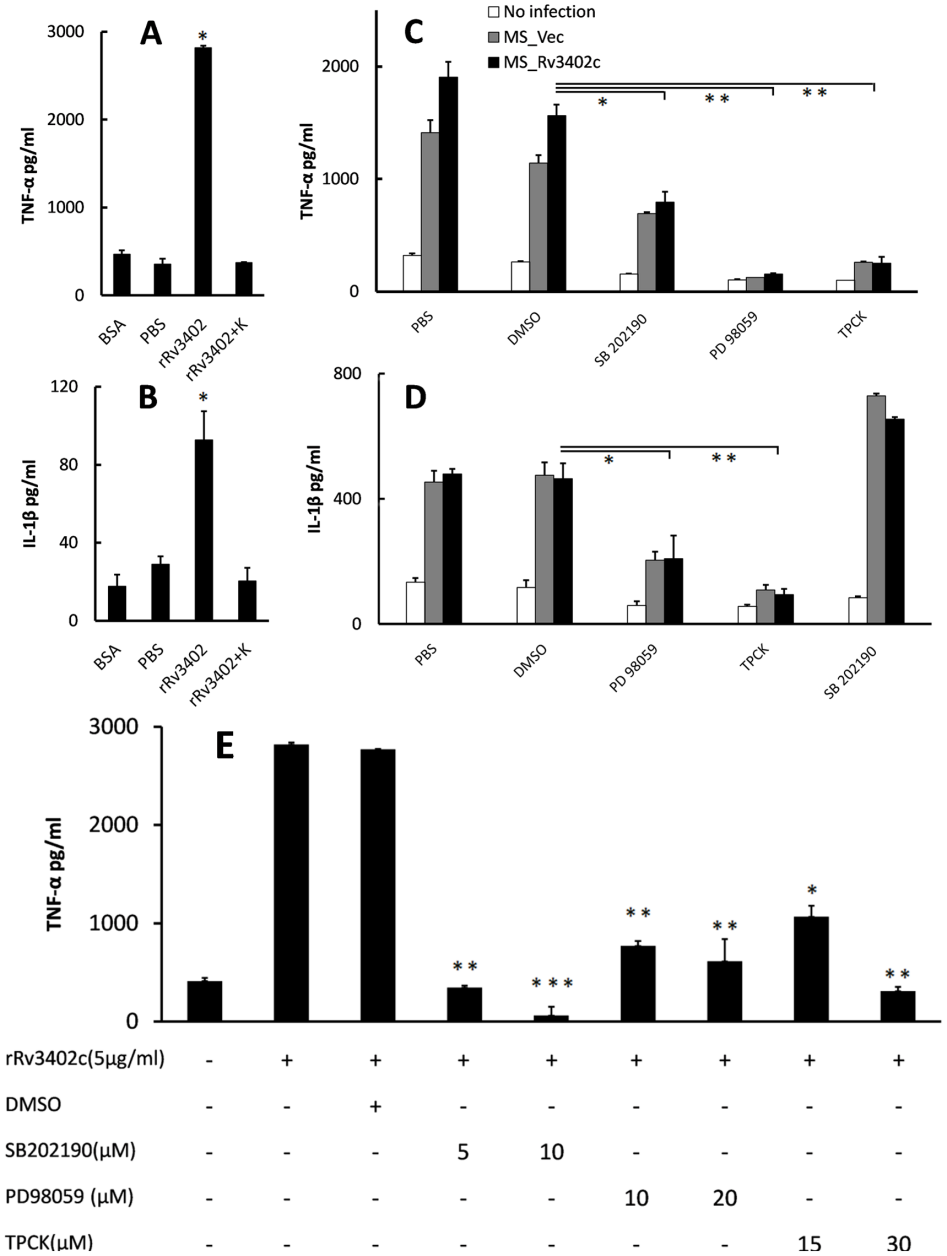
Rv3402c alters the activation levels of NF-κB, ERK and p38 in macrophages infected with *M. smegmatis*. Culture supernatants were harvested after 12 μg/ml and the concentrations of TNF-α (A) and IL-1β (B) were determined. U-937 cells were pre-treated with 30 μM TPCK (a NF-κB inhibitor) or with 20 μM PD 98059 (an ERK1/2 inhibitor) or with 10 μM SB 202190 (a p38 inhibitor). Treatment with DMSO served as a control for the inhibitor treatments. After 1 h, the macrophages were infected with MS_Vec at an MOI of 10 or treated with rRv3402c at the concentration of 5 μg/ml. Protease K (K) used to digest the recombinant protein. Culture supernatants were harvested after 24 h of infection and the concentrations of TNF-α (C, E) and IL-1β (D) were determined. (A), (B) and (E) are performed with recombinant protein; (C) and (D) are performed with *M. smegmatis* strains. The data are representative of two independent experiments.

To explore the potential role of Rv3402c in subverting the innate immune response, we investigated the levels of pro-inflammatory cytokines upon infection of U-937 with MS_Vec and MS_Rv3402c. Macrophages infected with MS_Rv3402c secreted significantly higher levels of TNF-α and IL-1β than the MS_Vec infected cells (Fig. 6A and C). To confirm these observations, the pro-inflammatory cytokine mRNA expression was determined using semi-RT-PCR. Compared to the MS_Vec, mRNA levels of TNF-α (Fig. 6B) and IL-1β (Fig. 6D) were significant upregulated in macrophages after 12 hours of infection with MS_Rv3402c. Similar results were obtained when U-937 cells were stimulated with rRv3402c (Fig. 7A and B). No difference was found for the levels of other inflammatory proteins, including IL-6, IL-10, and IL-12 p40 (data not shown). Collectively, our data suggests that Rv3402c might play a role in modulating the pro-inflammatory cytokine production of macrophages infected with *M. smegmatis*.

### Rv3402c robustly activates macrophage NF-κB, ERK and p38 signaling

NF-κB is a major transcription factor responsible for the transcription of TNF-α [36], [37], [38]. ERK 1/2 activation is essential for TNF-α production by macrophages [39], [40], and the MAPK pathways are crucial for macrophage signaling during mycobacterial infection [39], [41]. The increased TNF-α and IL-1β mRNA and protein expression (Fig. 6) raised the speculation that the activation of these signaling pathways might be altered within macrophages infected with MS_Rv3402c. To test this possibility, pharmacological inhibition experiments were used to confirm the requirement for NF-κB, ERK and p38 activities in the production of TNF-α and IL-1β in macrophages infected with MS_Rv3402c. MS_Rv3402c-induced TNF-α expression and protein production were significantly inhibited in U-937 cells pretreated with the specific NF-κB inhibitor TPCK, I kappa B-protease inhibitor [42], the ERK 1/2 inhibitor PD 98,059, and the p38 inhibitor SB 202190. However, IL-1β mRNA and protein expression were only inhibited by NF-κB specific inhibitor and the ERK 1/2 inhibitor (Fig. 7C and D). After stimulation with rRv3402c for the time points indicated, similar results were obtained (Fig. 7E). Pretreatment of U-937 cells with the NF-κB inhibitor, the ERK 1/2 inhibitor and the p38 inhibitor significantly reduced the production of TNF-α in a dose-dependent manner (Fig. 7E). Taken together, these results imply that the NF-κB, ERK and p38 pathways are required for Rv3402c-induced TNF-α and IL-1β production by macrophages.

## Discussion

The persistence of pathogenic mycobacteria within macrophages is in part due to the bacteria's ability to actively manipulate host signaling. Several mycobacterial secreted virulence factors have been well defined in the process of inhibiting phagosomal maturation, and such knowledge will unveil previously unknown host signaling pathways involved in the innate immunity against Mtb infection, which will lead to the development of more effective vaccines and drugs.

Our data suggests that the Rv3402c protein might play a role in intracellular survival of recombinant *M. smegmatis* by modulating the host innate immune response. *M. smegmatis*, a non-pathogenic mycobacterium species, does not multiply and is readily killed by the macrophage, enabling this bacteria to be an ideal surrogate for the identification of virulence factors implicated in intracellular survival [26], [32], [33], [43], [44], [45], [46], [47], [48], [49]. We found that *M. smegmatis* recombinant strain expressing Rv3402c showed enhanced survival both in the U-937 and the RAW264.7 cultures *in vitro* and impaired the viability of U-937. These results were not observed in the *M. smegmatis* strain containing the vector only. Since these two recombinant strains show the same growth kinetics in axenic culture or other stress conditions, the enhanced intracellular survival in macrophages of MS_Rv3402c was thought to be the result of the protein's interference with the innate immune response. Differences in viability between the U-937 and RAW264.7 cells infected with MS_3402c was possibly due to the inherent differences between mouse and human macrophages. For example, the Mtb *ptpA* deletion mutant was described to have reduced virulence in THP-1 cells [9], [10]. However, the same mutant was not found to have reduced survival in a mouse infection model [50]. Thus, definitive identification of the role of Rv3402c in these two models will require further functional studies which are beyond the scope of this study.

Despite recent evidence of the role of *rv3402c* in the interaction of recombinant *M. smegmatis* and host cells, the function of Rv3402c remains unknown. According to the TubercuList website (http://tuberculist.epfl.ch/index.html), *rv3402c* encodes a conserved hypothetical protein. Rv3402c is predicted to be engaged in the biosynthesis of lipopolysaccharide (LPS)-like molecules, and its sequence shows >40% sequence similarity to three types of enzymes: an aminotransferase, a dehydratase and an enzyme involved in perosamine/O-antigen biosynthesis [21]. Moreover, using the NCBI BLAST server we found that *rv3402c* encodes a protein with high sequence similarity to the lipopolysaccharide biosynthesis protein from *Bacillus cereus* and a putative PLP-dependent enzyme from *Rheinheimera sp*., which is predicted to be involved in cell wall biogenesis. We therefore tested whether Rv3402c was altered the cell surface properties. We found that the overexpression in *M. smegmatis* of Rv3402c, which is found in the cell envelope of mycobacteria, does not impact on colony morphology (data not shown). Furthermore, bacterial fatty acids compositions of *M. smegmatis* was not affected by the overexpression of Rv3402c protein (data not shown).

Although the physiologic function of Rv3402c is poorly understood, based on the subcellular fractionation analysis in this study, this protein deserved to be investigated for its role as a membrane- or cell wall-associated components. In order to avoid the bias of plasmid pNIT-Myc-Rv3402c, we chose another widely used plasmid pALACE to perform proteinase K and trypsin sensitivity assays. These assays performed on live recombinant mycobacterial strains revealed that His-tagged Rv3402c protein is partly exposed on the surface of mycobacteria. Not surprisingly, there are many proteins detected both in the cytoplasm and cell envelope, such as Rv2224c [51], Rv0132c [27], Eis [52] PknI [53], *et al*. Together, these results provide evidence that the Rv3402c protein is present on the surface of mycobacteria and may be available for interaction with host components.

TNF-α, a critical pro-inflammatory cytokine, is essential for host protective immunity to contain *M. tuberculosis* infection [54], [55]. Compared to pathogenic mycobacteria, *M. smegmatis* is a potent inducer of TNF-α in macrophages [56], consistent with the elevated TNF-α levels in the supernatant of macrophages infection with MS_Vec instead of the non-infected groups. Elevated levels of TNF-α was regarded as one culprit for Mtb persistence and virulence within human macrophages [45]. Previous studies have shown that mycobacterial components can enhance the production of TNF-α in monocytes/macrophages [45], [57], [58], [59]. Other studies have shown that virulent growth of Mtb in human monocytes [60], [61] or alveolar macrophages [62] is associated with enhanced secretion of TNF, as well as cytotoxicity. This evidence may provide insight into the role of TNF in cytolysis and the enhanced survival of the *M. smegmatis* strain expressing Rv3402c. Finally, through the pharmacological inhibition experiments, we found that the NF-κB, ERK and p38 pathways are required for Rv3402c-induced TNF-α expression by macrophages. However, only the NF-κB and ERK1/2 pathways are required for Rv3402c-induced IL-1β expression and protein production. It is our observation that Rv3402c disrupts host signal transduction in an alternative manner, which ultimately enables the bacteria to subvert the host immune response.

In summary, the present study suggests that the expression of the Rv3402c protein in *M. smegmatis* provides the bacteria specific properties that enhance intracellular persistence, decrease viability and modify the pro-inflammatory cytokine response of macrophages. This might provide novel information in the complex interplay between the host and Mtb. A greater understanding of the effect of Rv3402c on host signaling pathways will enhance our knowledge of Mtb pathogenesis. Further experiments such as using Mtb knockout mutant to determine the precise function of Rv3402c during infection are worthwhile.
