# Correction: *Mycobacterium tuberculosis* Rv3402c Enhances Mycobacterial Survival within Macrophages and Modulates the Host Pro-Inflammatory Cytokines Production via NF-Kappa B/ERK/p38 Signaling

**DOI:** 10.1371/journal.pone.0113942

**Published:** 2014-11-12

**Authors:** The PLOS ONE Staff

There is an error in the second sentence of the “Intracellular survival assay” subsection of the Materials and Methods. The correct sentence is: Following 48 hours of treatment with 0.1 µg/ml of phorbol 12-myristate 13-acetate (PMA) (Sigma), U-937 cells were transformed into an adherent state.
